# Ceruloplasmin functional changes in Parkinson’s disease-cerebrospinal fluid

**DOI:** 10.1186/s13024-015-0055-2

**Published:** 2015-11-04

**Authors:** Marco Barbariga, Flavio Curnis, Annapaola Andolfo, Alan Zanardi, Massimo Lazzaro, Antonio Conti, Giuseppe Magnani, Maria Antonietta Volontè, Laura Ferrari, Giancarlo Comi, Angelo Corti, Massimo Alessio

**Affiliations:** Proteome Biochemistry, IRCCS-San Raffaele Scientific Institute, via Olgettina 60, 20132 Milan, Italy; Tumor Biology and Vascular Targeting, IRCCS-San Raffaele Scientific Institute, via Olgettina 60, 20132 Milan, Italy; ProMiFa-Protein Microsequencing Facility, IRCCS-San Raffaele Scientific Institute, via Olgettina 60, 20132 Milan, Italy; INSPE-Institute of Experimental Neurology, IRCCS-San Raffaele Scientific Institute, via Olgettina 60, 20132 Milan, Italy; Vita-Salute San Raffaele University, via Olgettina 60, 20132 Milan, Italy; Present address: Translational Neurology group, Wallenberg Neuroscience Center, BMC A10, 221 84 Lund, Sweden

**Keywords:** Ceruloplasmin, Parkinson, Oxidative stress, Deamidation, Integrin-binding, Ferroxidase, NGR and *iso*DGR motif, Hydrogen peroxide

## Abstract

**Background:**

Ceruloplasmin, a ferroxidase present in cerebrospinal fluid (CSF), plays a role in iron homeostasis protecting tissues from oxidative damage. Its reduced enzymatic activity was reported in Parkinson’s disease (PD) contributing to the pathological iron accumulation. We previously showed that ceruloplasmin is modified by oxidation *in vivo*, and, in addition, *in vitro* by deamidation of specific NGR-motifs that foster the gain of integrin-binding function. Here we investigated whether the loss of ceruloplasmin ferroxidase activity in the CSF of PD patients was accompanied by NGR-motifs deamidation and gain of function.

**Results:**

We have found that endogenous ceruloplasmin in the CSF of PD patients showed structural changes, deamidation of the ^962^NGR-motif which is usually hidden within the ceruloplasmin structure, and the gain of integrin-binding function. These effects occur owing to the presence of abnormal levels of hydrogen peroxide we detected in the CSF of PD patients. Interestingly, the pathological CSF's environment of PD patients promoted the same modifications in the exogenously added ceruloplasmin, which in turn resulted in loss of ferroxidase-activity and acquisition of integrin-binding properties.

**Conclusions:**

We show that in pathological oxidative environment of PD-CSF the endogenous ceruloplasmin, in addition to loss-of-ferroxidase function, is modified as to gain integrin-binding function. These findings, beside the known role of ceruloplasmin in iron homeostasis, might have important pathogenic implications due to the potential triggering of signals mediated by the unusual integrin binding in cells of central nervous system. Furthermore, there are pharmacological implications because, based on data obtained in murine models, the administration of ceruloplasmin has been proposed as potential therapeutic treatment of PD, however, the observed CSF's pro-oxidant properties raise the possibility that in human the ceruloplasmin-based therapeutic approach might not be efficacious.

**Electronic supplementary material:**

The online version of this article (doi:10.1186/s13024-015-0055-2) contains supplementary material, which is available to authorized users.

## Background

Ceruloplasmin (Cp) is a ferroxidase enzyme expressed on the surface of astrocytes and secreted as a soluble form in the cerebrospinal fluid (CSF) by choroid plexus epithelial cells [1]. Ceruloplasmin plays an important role in cellular iron homeostasis and protects tissues from oxidative damage [2, 3]. Significant loss of ceruloplasmin-ferroxidase activity has been observed in CSF and substantia nigra of patients with Parkinson's disease (PD), suggesting a role of ceruloplasmin dysfunction in neurodegenerative diseases characterized by oxidative stress [3–7]. Consistently, ceruloplasmin-KO mice and patients with genetic aceruloplasminemia have increased iron accumulation in brain, neurodegeneration and motor deficits [7–10]. It has been shown in a PD-mouse model that administration of ceruloplasmin can attenuate neurodegeneration and thus, it has been proposed that infusion of ceruloplasmin to PD patients might be of therapeutic utility [7, 11].

The loss of ceruloplasmin-ferroxidase activity observed in PD patients might be the consequence of the protein oxidation we observed [3] likely owing to redox changes in pathological CSF [12, 13]. We also have previously shown that ceruloplasmin oxidation may promote asparagine (Asn, N) deamidation, a spontaneous reaction that occurs during protein aging and that leads to aspartate (Asp, D) and isoaspartate (*iso*Asp, *iso*D) formation [14]. In particular, two residues of ceruloplasmin (^568^N and ^962^N), that are part of Asparagine-Glycine-Arginine-(NGR)-sequences, can deamidate with a fast kinetics [14] leading to the formation of *iso*DGR, a motif capable of recognizing the Arginine-Glycin-AsparticAcid-(RGD)-binding site of integrins [15, 16]. Accordingly, *in vitro* NGR-deamidation in ceruloplasmin is associated with gain of integrin-binding and intracellular signalling functions, that through FAK1, ERK1/2, Akt and MAPK involvement may regulate gene activation, cell cycle, proliferation, and actin cytoskeleton rearrangement [14]. While ^568^N is exposed on the protein surface and can rapidly deamidate *in vitro*, deamidation of ^962^N requires protein oxidation and structural changes, being this residue buried within the protein tertiary structure [14]. These notions prompted us to investigate whether the loss of ferroxidase activity in PD patients is accompanied by NGR-to-isoDGR transition. We show here that the endogenous ceruloplasmin in the CSF of PD patients exhibited structural changes, deamidation of the ^962^NGR-motif and gain of integrin binding function, owing to the presence of abnormal levels of hydrogen peroxide in the CSF. Interestingly, the pathological environment in the CSF of PD patients promotes the same modifications in the exogenously added ceruloplasmin, with the consequent loss of ferroxidase activity and gain of integrin binding properties, which in turn rise warnings about a potential therapeutic use of ceruloplasmin in patients.

## Results

### The endogenous ceruloplasmin in the CSF of PD patients exhibits structural changes, deamidation of the ^962^NGR-motif and gain of integrin binding function

**Fig. 1:**
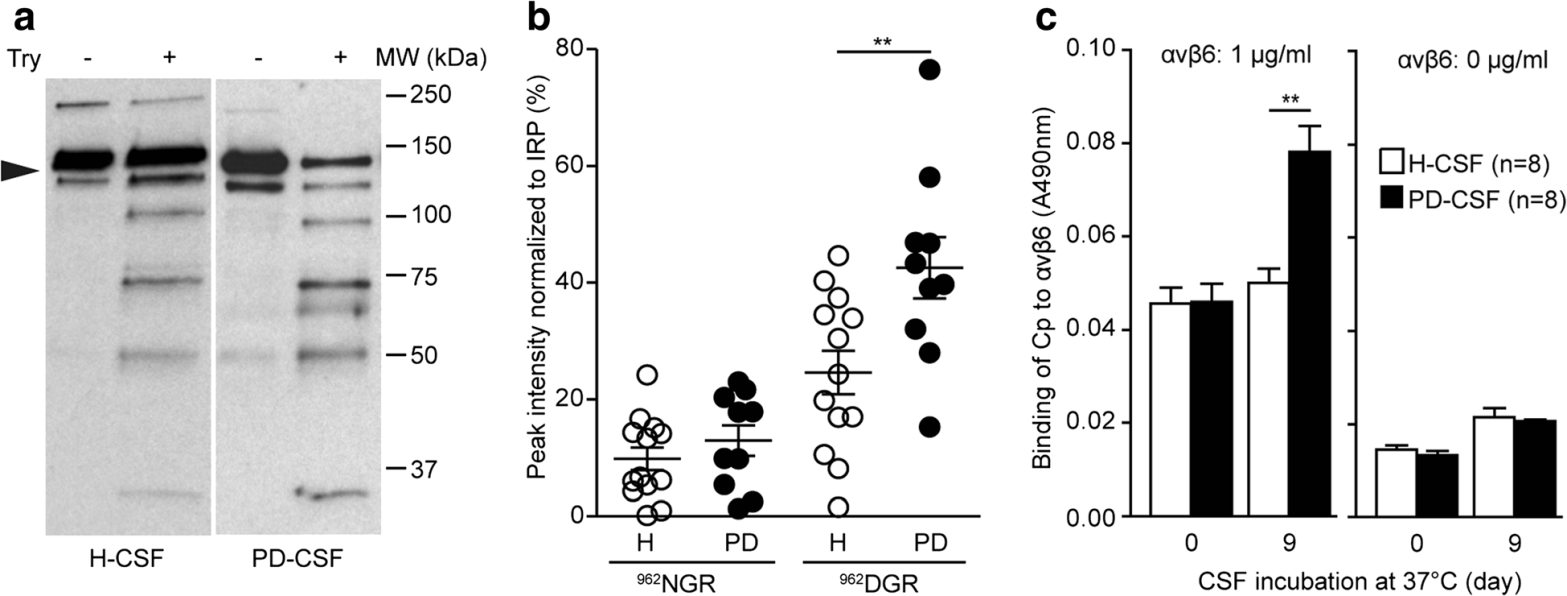
Ceruloplasmin in PD patients CSF shows structural changes, ^962^NGR-motif deamidation and gain of integrin-binding function. **a**) Structural changes. Western blot analysis after limited trypsin proteolysis (Try, +) showed higher sensitivity of the endogenous ceruloplasmin from PD’s CSF compared to Cp from healthy subjects (H-CSF). **b**) Deamidationof the ^962^NGR-motif. CSF samples (70 μl each) from healthy subjects (H, n = 13) and PD patients (PD, n = 10) were digested with trypsin and directly analysed by quantitative parallel reaction monitoring mass spectrometry. Data are reported as peak-intensity of the peptide containing the ^962^NGR-motif as it is (MHAINGR) and deamidated (MHAIDGR), normalized for the ceruloplasmin internal reference peptide (IRP), namely LISVDTEHSNIYLQNGPDR, which yielded the lowest median CV for both ^962^NGR- and ^962^DGR-containing peptides. Five technical replicates for each sample were run and use for the quantification. **c**) Binding to αvβ6 of endogenous ceruloplasmin from CSF of healthy subjects (H-CSF) or PD patients (PD-CSF) revealed by ELISA at time zero or after 9 days aging. Two independent experiments in triplicate were performed using CSFs of different subjects in each experiment (total subjects for each group n = 8). In B and C data were analyzed by student’s *t* test; means with standard error, calculated using pooled data from different experiments, are indicated (** = p < 0.01)

To investigate whether the oxidative modifications we observed in the ceruloplasmin from the CSF of PD patients [3] were also associated with structural changes that may foster ^962^NGR- to isoDGR-motif transition, as reported for ceruloplasmin *in vitro* [14], we performed a limited trypsin proteolysis assay. The endogenous ceruloplasmin in the CSF from PD patients showed higher sensitivity to proteolysis than did that in the CSF from healthy subjects (Fig. 1a), indicating the structural changes occur in the patients' CSF. This finding suggested that deamidation of ^962^NGR-motifs may take place *in vivo* in the patients. Quantitative mass spectrometry (MS) analysis performed by parallel reaction monitoring (PRM) on the CSF of healthy subject (H-CSF, *n* = 13) and PD patients (PD-CSF, *n* = 10) confirmed that the deamidation of the endogenous ceruloplasmin ^962^NGR-motif was significantly higher (*p* = 0.0094) in PD patients compared to healthy subjects (Fig. 1b). On the contrary to the ^962^NGR-motif, the ^568^NGR-motif was poorly- or not-deamidated in both control and PD patients (data not shown). No correlations have been found between the rate of Cp-deamidation and the demographic/clinical parameters evaluated in patients (age, disease duration, disease score), nor correlation has been found with the samples' time of storage at −80 °C (not shown). Considering the physiological ceruloplasmin concentration of about 1.5 μg/ml in the CSF [17], the increased deamidation of about 18 % found in PD-CSF compared to H-CSF (Fig. 1b), corresponded to about 270 ng/ml of deamidated ceruloplasmin. Furthermore, considering that the deamidated peptide detected by mass spectrometry is representative of both ^962^DGR and ^962^*iso*DGR forms, and that only the latter is responsible for the integrin-binding, we can hypothesize, according to the reported 50-75 % conversion of NGR toward *iso*DGR [15], that the amount of deamidated ceruloplasmin capable of integrin recognition ranges from 135 to 200 ng/ml. Therefore, we then analysed the integrin-binding properties of the endogenous ceruloplasmin. We tested the binding activity to the αvβ6-integrin since we already reported the specific binding capability of the *in vitro* oxidized/deamidated ceruloplasmin to this integrin [14]. No specific binding to αvβ6-integrin was observed for endogenous ceruloplasmin from both healthy subjects and PD patients under resting conditions (Fig. 1c). This finding was probably due to the lower sensitivity of the ELISA compared to MS analysis, therefore, it is likely that the scarcity of adhesive-ceruloplasmin (containing the *iso*DGR) available determined the capture and detection of this protein in very small amounts. Moreover, competition with other integrin-binding molecules present in CSF (e.g. fibronectin, tenascin) was a further constraint on adhesive-ceruloplasmin capture. Interestingly, if the CSFs were aged *in vitro* for 9 days, a significant αvβ6-integrin binding activity (*p* = 0.0023, CV <13 %) was induced exclusively in the CSF from PD patients (Fig. 1c) suggesting that the pathological environment can promote a gain-of-integrin binding activity, likely mediated by ^962^NGR deamidation and *iso*DGR formation. These findings indicated that ceruloplasmin deamidation of NGR-motifs occurs *in vivo* in the patients CSF and suggested that the ^962^NGR-motif plays a major role in the acquisition of the integrin binding properties.

### The CSF from PD patients promotes spiked ceruloplasmin structural changes that foster loss of ferroxidase activity and ^962^NGR-deamidation

**Fig. 2:**
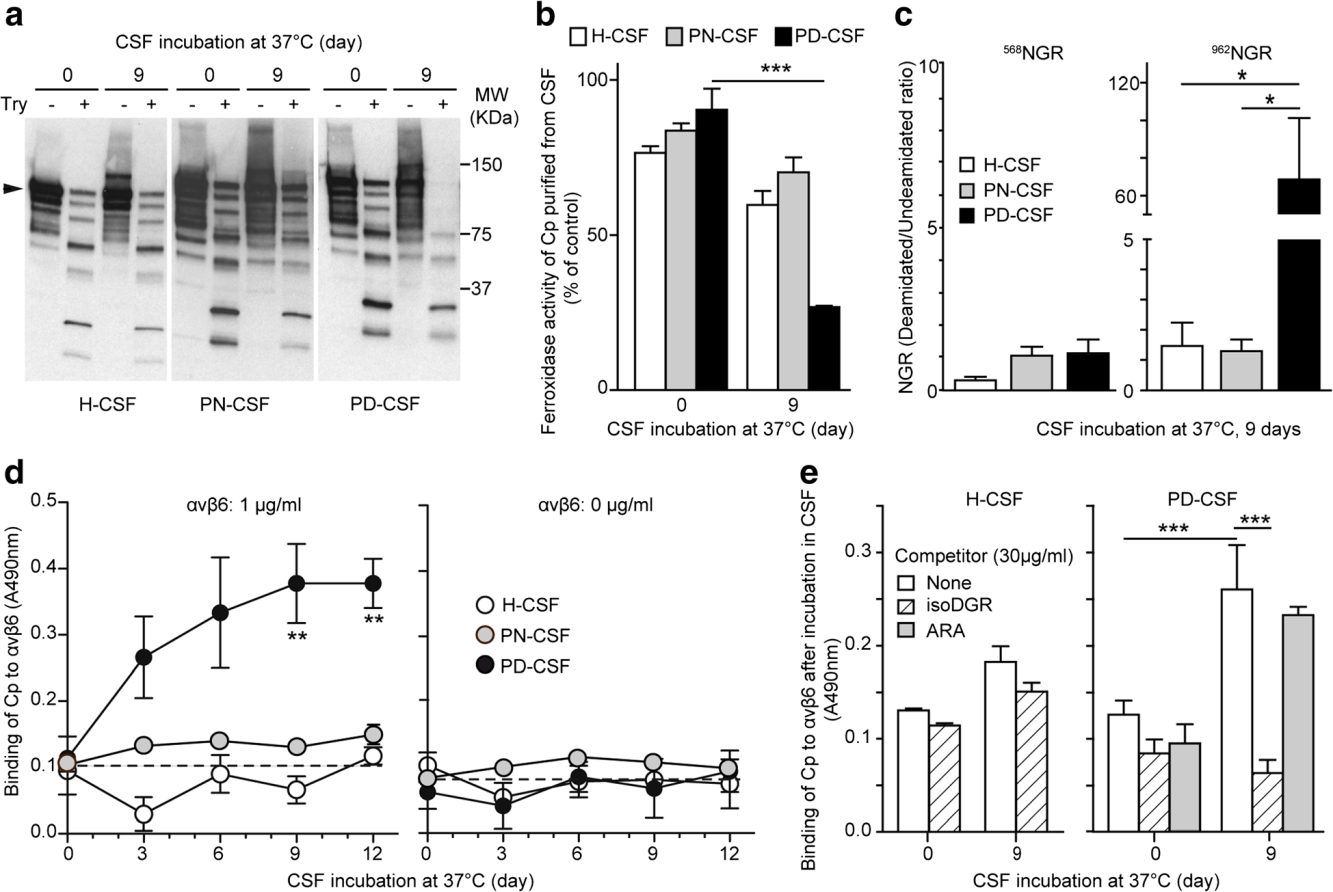
Incubation of ceruloplasmin spiked in the CSF of PD patients induces structural and functional modifications. Ceruloplasmin (Cp) was added (20 μg/ml) to CSF from healthy subjects (H-CSF), peripheral neuropathies patients (PN-CSF) and PD patients (PD-CSF), and incubated for 0 or 9 days at 37 °C, then supplemented CSFs were directly used or ceruloplasmin was immunoprecipitated and used for different assays. **a)** Structural changes. Immunoprecipitated samples were subjected to limited-trypsin digestion and analyzed by western blot with an anti-ceruloplasmin Ab (a representative experiment is shown). **b**) Ferroxidase activity. The activity of immunoprecipitated Cp (1.25 μg) was evaluated by bathophenanthroline assay and expressed as % of ferroxidase activity measured for 1.25 μg of untreated ceruloplasmin. Three independent experiments in triplicate were performed using CSFs from different subjects in each experiment (total subjects for each group n = 8). **C)** Deamidation of NGR-motifs. Ceruloplasmin immunoprecipitated from CFSs samples after 9 days of incubation, was trypsin-digested and analysed by mass spectrometry. Semi-quantitative label-free evaluation was performed on the observed peptides containing either ^568^NGR- or ^962^NGR-motif as they are or deamidated. Data are reported as ratio between deamidated and non-deamidated ^568^NGR or ^962^NGR quantitation; three independent experiments were performed using CSF from different subjects each group (total subjects for each group n = 12; in PN-group the PN1 sample was used twice). **d-e)** Binding to αvβ6-integrin of ceruloplasmin after different time of incubation (0–12 days at 37 °C) in different CSFs was evaluated by direct (**d**) or competitive (**e**) αvβ6-integrin binding ELISA assay using the CSFs supplemented with ceruloplasmin. Competition experiments were performed using either acetyl-CisoDGRCGVRSSSRTPSDKY (isoDGR) peptide or the control peptide acetyl-CARACGVRSSSRTPSDKY (ARA). Two independent experiments in triplicate were performed using CSFs from different subjects in each experiment (total subjects for each group *n* = 8). In all assays data were analyzed by student’s *t* test; means with standard error, calculated using pooled data from different experiments, are indicated (*** = p < 0.001; ** = p < 0.01; * = p < 0.05)

In order to confirm that the environment of CSF from PD patients can promote ceruloplasmin modifications, we added purified ceruloplasmin to the CSF of healthy subjects (H) or patients with peripheral neuropathies (PN) or PD, and analysed it before and after incubation for different times. Ceruloplasmin aged for 9 days in PD-CSF, but not in H-CSF or PN-CSF, showed a higher sensitivity to limited trypsin proteolysis and a significant reduction of ferroxidase activity (*p* = 0.0011) (Fig. 2a and b). These results indicated the occurrence of structural changes that may be responsible for ceruloplasmin enzymatic activity reduction as previously reported [3, 14, 18]. Semiquantitative MS analysis of spiked ceruloplasmin-derived peptides after incubation in PD-CSF was performed to characterize the deamidation of NGR-sites. Similarly to what was observed for the endogenous ceruloplasmin, the deamidation of ^962^NGR, but not of ^568^NGR, was significantly enhanced, compared to both controls H-CSF and PN-CSF, after 9 days of incubation in PD-CSF (*p* = 0.0164) (Fig. 2c). These findings support the hypothesis that structural changes occur in ceruloplasmin after incubation in PD-CSF, which are likely crucial for both loss of ferroxidase activity and cryptic ^962^NGR/*iso*DGR-site exposure.

### The CSF from PD patients promotes spiked ceruloplasmin gain of integrin-binding function mediated by isoDGR-motif/s

We analysed the ceruloplasmin integrin-binding properties before and after incubation in CSFs. Ceruloplasmin aging in PD-CSF, but not in H-CSF or PN-CSF, could induce αvβ6-integrin binding activity (*p* = 0.0091 and *p* = 0.0083 at 9 and 12 days, respectively) (Fig. 2d). The binding was competed by an excess of *iso*DGR-peptide (*p* = 0.0009), but not by a control ARA-peptide (Fig. 2e), indicating that the environment of PD-CSF can promote a gain-of-integrin binding activity, likely mediated by ^962^NGR deamidation and *iso*DGR formation.

### Oxidative stress contributes to Cp-structural changes

**Fig. 3:**
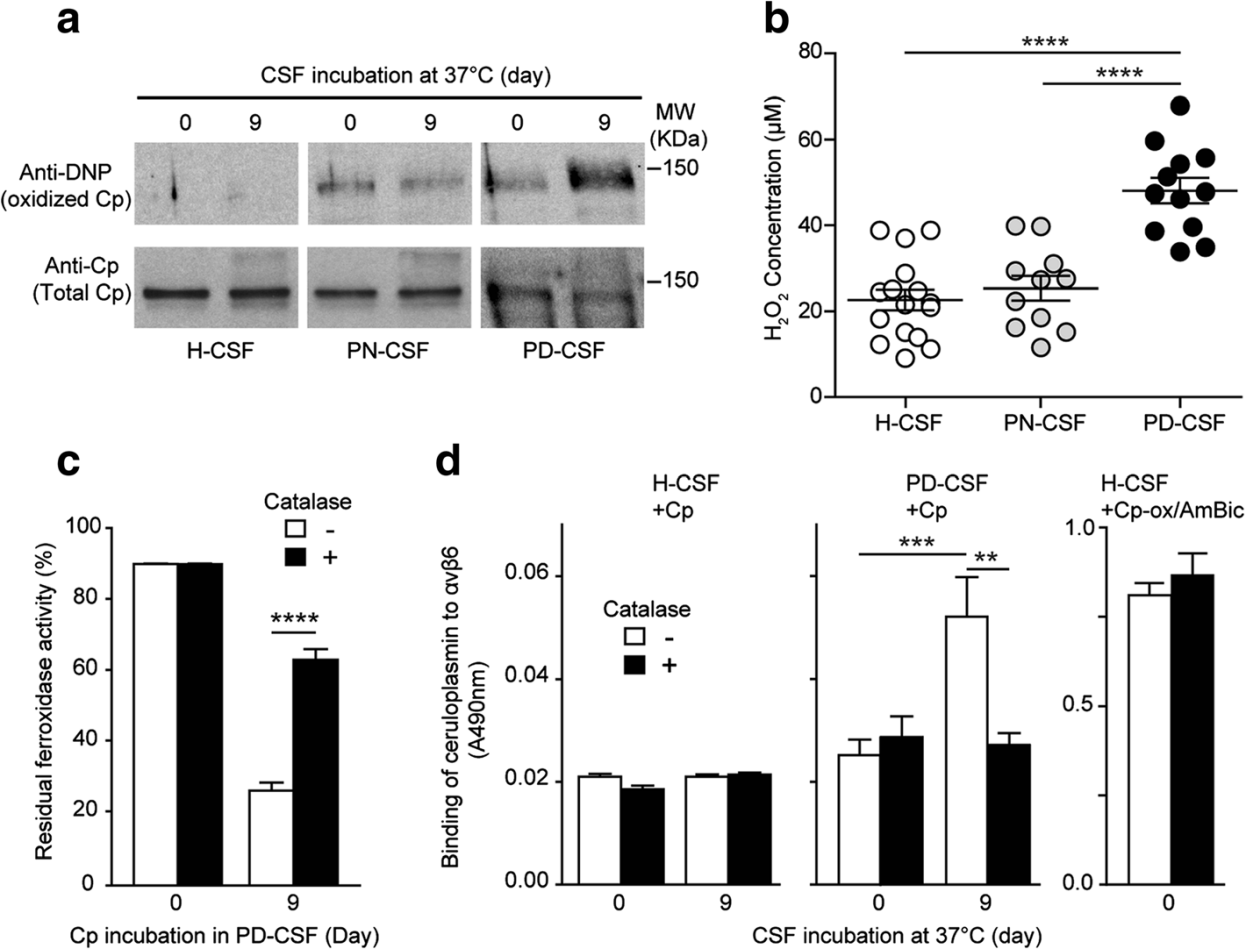
Oxidative stress contributes to ceruloplasmin structural changes and gain of integrin-binding function. **a**) Analysis of ceruloplasmin (Cp) oxidation after incubation in H-CSF, PN-CSF, or PD-CSF. Cp was added to CSFs (20 μg/ml), incubated (37 °C) for 0 or 9 days, and immunoprecipitated. One part of the product was analyzed for protein oxidation rate using the OxyBlot™ kit (based on anti-DNP WB analysis of protein carbonylation), and the other part was analyzed by WB with anti-ceruloplasmin Ab. Two independent experiments were performed using pools of CSFs from 4 different subjects per groups in each experiment (total subjects analyzed for each group n = 8) (a representative experiment is shown). **b**) Quantification of hydrogen peroxide concentration in H-CSF (n = 16), PN-CSF (n = 11) and PD-CSF (n = 12) using the Amplex® Red Hydrogen Peroxide kit. **c)** Effect of hydrogen peroxide depletion with catalase on the ceruloplasmin loss-of-ferroxidase activity induced by incubation in PD-CSF. Ceruloplasmin (20 μg/ml) and catalase (60 μg/ml) were added to H-CSF or PD-CSF, left to incubate (37 °C) for 0 or 9 days, then the ferroxidase activity of immunoprecipitated ceruloplasmin (1.25 μg) was evaluated by bathophenanthroline assay and expressed as % of ferroxidase activity measured for 1.25 μg of untreated ceruloplasmin. **d**) Effect of catalase-mediated hydrogen peroxide depletion on the ceruloplasmin gain of integrin-binding properties induced by incubation in PD-CSF. Ceruloplasmin and catalase were added to different CSFs as in C and analyzed by direct αvβ6-binding assay. To rule out a non-specific effect of catalase on ceruloplasmin-integrin-binding, a control with ceruloplasmin pre-oxidized and deamidated *in-vitro* (Cp-ox/AmBic) (right panel) was performed. In **c** and **d**, two independent experiments in triplicate were performed using CSFs from different subjects in each experiment (total subjects for each group *n* = 8). In all experiments statistical p value was evaluated by student’s *t* test, means with standard error, calculated using pooled data from different experiments, are indicated (**** = p < 0.0001 *** = p < 0.001, ** = p < 0.01)

To assess the contribute of oxidative stress in structural and functional changes observed after ceruloplasmin aging in PD-CSF, we analysed ceruloplasmin carbonylation before and after 9 days of incubation, as read-out of protein oxidation promoted by reactive oxygen species. Western blot (WB) analysis showed a weak carbonylation at time 0 in PN-CSF and PD-CSF, but not in H-CSF (Fig. 3a). Remarkably, ceruloplasmin aging in PD-CSF, but not in H-CSF or PN-CSF, induced an increase of protein carbonylation (Fig. 3a), pointing to oxidative stress in this condition.

Interestingly, the PD-CSF showed a significant higher concentration of hydrogen peroxide (48 μM) compared to H-CSF or PN-CSF (p < 0.0001) (21.9 and 24.9 μM, respectively) (Fig. 3b). This observation suggests a role for hydrogen peroxide in ceruloplasmin oxidation and consequent structural and functional changes. Accordingly, degradation of hydrogen peroxide by catalase addition at the beginning of incubation, prevented the reduction of ceruloplasmin ferroxidase activity observed after 9 days of incubation in PD-CSF (p < 0.0001) (Fig. 2b and Fig. 3c). Noteworthy, the presence of catalase also prevented the gain of integrin-binding activity of ceruloplasmin observed after 9 days of incubation in PD-CSF (*p* = 0.009) (Fig. 3d). Of note, catalase could not reduce the integrin-recognition of ceruloplasmin pre-oxidized/deamidated *in vitro* (Cp-ox/AmBic) and added to H-CSF (Fig. 3d), indicating that catalase was not able to revert the oxidative modifications already induced, therefore sustaining the evidence that catalase acts scavenging the hydrogen peroxide present in PD-CSFs.

These findings support the hypothesis that hydrogen peroxide was crucially involved in ceruloplasmin oxidation and in the induction of structural changes necessary for loss of ferroxidase activity, ^962^NGR-deamidation and gain of integrin-binding properties.

## Discussion

In this study we demonstrated that, in addition to the oxidative modifications and the loss of ferroxidase activity already reported [3–7], the ceruloplasmin in the CSF of PD patients undergoes to conformational changes and NGR-motifs deamidation, which in turn promote the gain of integrin-binding function. *In vivo* NGR-deamidation, on the contrary to what was observed *in vitro* [14], occurs primarily at the hidden ^962^NGR-site and not, or markedly less, on the surface-exposed ^568^NGR. However, this finding is conceivable because asparagine deamidation depends on neighboring residues as well as on secondary and tertiary structure of the protein, and is also affected by environmental factors such as ionic strength and pH [19, 20]. The kinetic of deamidation reactions is influenced by the combination of these factors, thus can range from hours to years for different residues under different conditions [19, 20]. As ^962^NGR-deamidation requires a conformational change to occur [14], this implies that structural changes were induced in PD-CSF ceruloplasmin, likely consequent to the increased oxidation of CSF’s protein as reported in PD [3, 21]. *In vivo*, asparagine deamidation has been reported in pathological conditions associated with oxidative stress as for example for Tau protein and β-amyloid peptides in Alzheimer's disease [22–25]. Thus the ceruloplasmin’s modification fostered by pathological environment we described here might explain the loss of ceruloplasmin-ferroxidase activity reported in CSF and substantia nigra of patients with PD [3–7]. This might have relevant pathological implications in the brain cellular iron homeostasis with consequent increase in iron accumulation, oxidative damage and neurodegeneration.

We have previously shown that *in vitro* deamidation of ceruloplasmin's NGR-motifs promotes the gain of integrin-binding function, and that the interaction of deamidate ceruloplasmin with integrins resulted in an intracellular signal transduction that affects cell physiology [3, 14]. The absence of binding to αvβ6-integrin we found for endogenous ceruloplasmin from PD patients under resting conditions might depend on both, the very low amount of deamidated ceruloplasmin suitable for the binding (namely the *iso*DGR isoform), and the sensitivity of the method used for binding detection. In addition, we also hypothesized that, *in vivo,* as soon as the ceruloplasmin is modified, and thus becomes able to bind the integrins, it is sequestered by the surrounding, integrin-expressing tissues. In fact, *in vitro* in the absence of sequestering integrin-expressing tissue, the CSF from PD patients aged for 9 days showed the acquisition of the αvβ6-integrin binding activity. Interestingly the gain of function was induced exclusively in the CSF from PD patients and not in the control CSFs from healthy subjects and PN patients. This rule out the induction of methodological-artificial oxidative conditions and suggested that the PD pathological environment was able to promote a gain-of-integrin binding activity. Whether the ceruloplasmin gain of integrin-binding function observed in PD-CSF has favourable or detrimental effects on disease progression is difficult to predict at present and need further investigations. However, since we reported that integrin binding by deamidated ceruloplasmin is able to activate *in vitro* an intracellular signaling cascades, which in turn could affect cell proliferation and cytoskeletal re-organization [14], it is conceivable to hypothesize that the deamidated ceruloplasmin found in PD patients might transduce unusual intracellular signals. Interestingly, an aberrant signaling mediated via integrin engagement by fibrillar amyloid-β, which resulted in neuronal dystrophy and death, has been reported in Alzheimer's disease [26–28]. Several cell types in the central nervous system might be potential targets of deamidated ceruloplasmin, including microglial cells and the specialized epithelial cells of the ependymal layer and choroid plexus, the latter being directly in contact with the CSF.

The second major finding of this work is that the CSF of PD patients contains elements capable of promoting structural and functional modifications in ceruloplasmin, including, oxidation/carbonylation, loss of ferroxidase activity, asparagine deamidation at the NGR-site and acquisition of integrin-binding properties. Indeed, NGR-deamidation of spiked ceruloplasmin was fostered *ex vivo* in PD-CSF at higher rates than in normal-CSF and PN-CSF used as control neurological disease. As for the endogenous ceruloplasmin, the deamidation of spiked ceruloplasmin occurs primarily at the hidden ^962^NGR-site, which implies the requirement of conformational changes as supported by the different sensitivity to limited proteolysis observed. These evidences suggest as a logical consequence that PD-CSF contains compounds capable of fostering these changes. Of note, we found that the PD-CSF contains high levels of hydrogen peroxide, a highly reactive oxygen species, and that catalase, an enzyme that converts hydrogen peroxide into water, prevented both the inhibition of ferroxidase activity and the acquisition of ceruloplasmin integrin-binding properties. Therefore, hydrogen peroxide known to be overproduced in neurodegeneration [29], seems to be an agent crucially involved in the structural and functional modifications of ceruloplasmin in PD-CSF. Dealing with metal ions, namely copper and iron, ceruloplasmin results to be very susceptible to environment redox-changes. However, in addition to the ceruloplasmin prototype, several others proteins might be modified by the pathological pro-oxidant environment in the CSF of PD patients, as inferred by the greater level of total protein carbonylation reported in the CSF of PD patients [3, 21]. These oxidative modifications may have consequences on proteins functions, like for example the reported inhibition of the extracellular superoxide dismutase enzymatic activity induced by the hydrogen peroxide [30]. Interestingly, it has been reported a higher propensity of α-synuclein (α-syn) to undergo deamidation, compared to β-syn, a modification that favor the formation of stable oligomers [31]. Thus, protein deamidation might foster the α-syn aggregate formation in the CSF of PD patients, where α-syn oligomers have been described [32–34]. Similarly a deamidation-mediated protein aggregation it has been reported for Tau and β-amyloid proteins in Alzheimer’s disease [23–25]. The oligomers in the CSF might contribute to the disease spreading according to the recently proposed prion concept for aggregated proteins both in Alzheimer’s and Parkinson’s diseases [35]. Furthermore, the high levels of hydrogen peroxide found in the CSF of PD patients might have additional pathological implications being this molecule able to affect several cellular functions [36] (reviewed in [37]). Another important question raised by the high level of hydrogen peroxide observed in the PD-CSF is how this compound is produced and/or accumulated in the CSF. An hypothesis is that, the pathological features of PD like aberrant autophagy and mitochondrial dis-functions that have been reported to potentiate the production of hydrogen peroxide [38, 39], might be responsible for the observed concentration increase.

The faster kinetic of ceruloplasmin’s NGR-motifs deamidation and loss of ferroxidase activity observed during *ex vivo* aging in PD-CSF compared to both H-CSF and PN-CSF supports the hypothesis of a generally accelerated protein aging in neurodegenerative diseases promoted by the pathological environment [12, 13]. The observation that approximately 50-60 % of the ceruloplasmin ferroxidase activity was lost after 9 days of aging in the PD-CSF, and the fact that the ceruloplasmin concentration in the CSF is about 1.5 μg/ml with a physiological half-life of about 5.5 days *in vivo* [1, 17], made conceivable that ceruloplasmin modifications might occur in patients to an extent that yields relevant concentration of non-functional protein, about 200 ng/ml which is in line with the concentration rage also suggested by PRM-MS results.

**Fig. 4:**
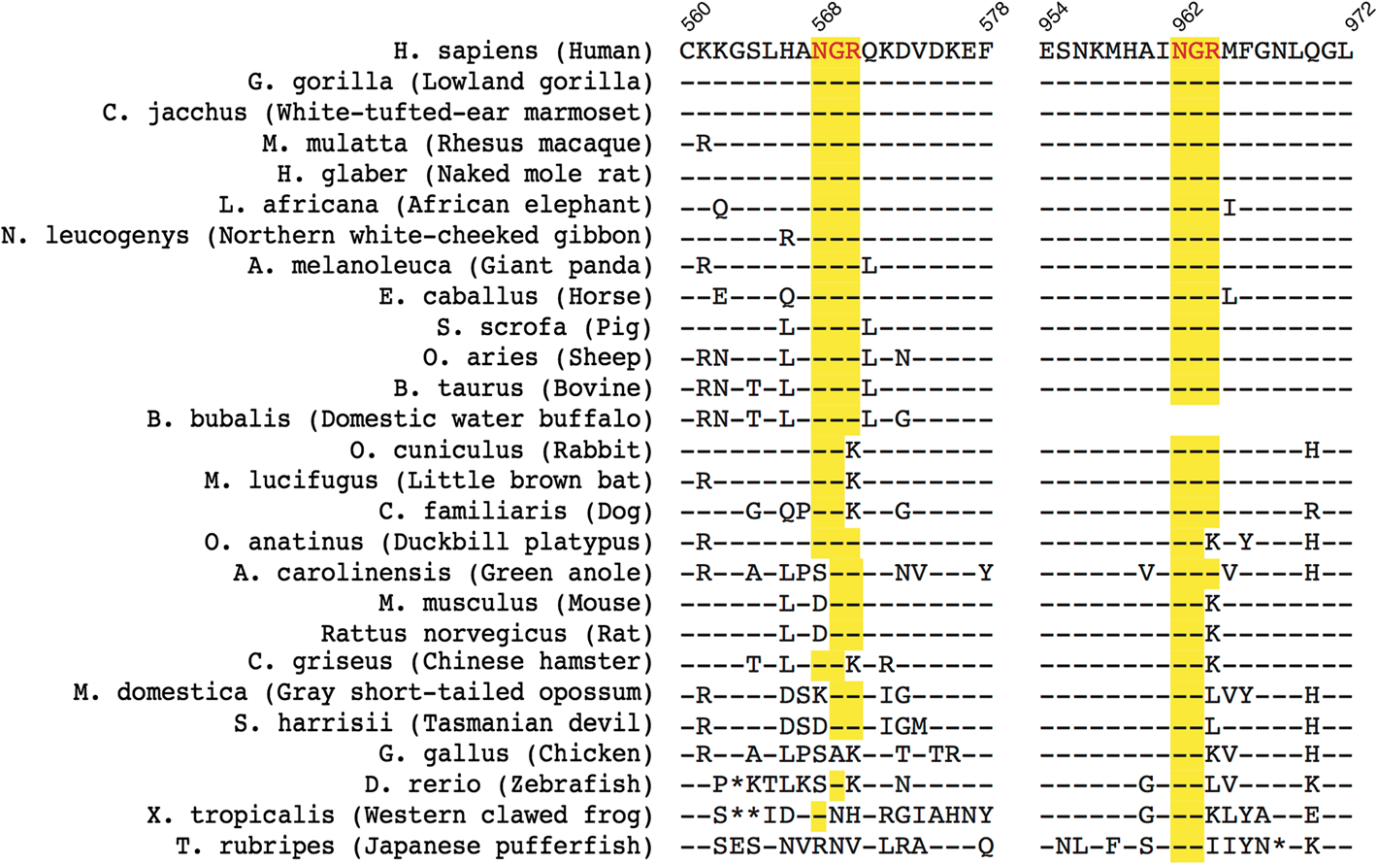
Analysis of the conservation of the Cp’s NGR motifs across different species. Sequence alignment of NGR-motifs present in ceruloplasmin from differing species was performed with ClustalX software (Conway Institute UCD, Dublin; www.clustal.org). The ^568^NGR- and ^962^NGR-motifs are conserved along different species, however, in mouse and rat the conservation is not maintained

Our findings showing that the pathological CSF is able to modify the ceruloplasmin arise warnings about a potential therapeutic use of this protein in patients [7], especially in the light of loss of ferroxidase activity promoted by the PD-CSF environment. Despite the promising results obtained in murine models treated with ceruloplasmin, it is presently unknown whether the CSF of PD-mice can induce oxidative dysfunctions as observed with human PD-CSF. Notably, in murine models ceruloplasmin-activity’s reduction is associated with protein expression reduction, whereas in PD-patients the activity reduction occurs in the presence of normal level of ceruloplasmin-expression suggesting different mechanism of ferroxidase activity impairment [7]. Furthermore, the high conservation of the ^568^NGR- and ^962^NGR-motifs across different species is not maintained in mouse and rat; in these species the sequences are ^568^DGR- and ^962^NGK- respectively, the first not prone to isomerize to *iso*DGR- and the second not described to be able to generate an integrin binding-motif when deamidated (Fig. 4). Therefore, the risk exists that data obtained in animal models might not completely predict the behaviour in PD patients.

## Conclusions

Our results show that, owing an aberrant hydrogen peroxide concentration, the pathological oxidative environment in PD-CSF is able to promote endogenous ceruloplasmin loss-of-ferroxidase function and gain-of-integrin binding activity. These effects might have important pathogenic implications affecting ceruloplasmin-mediated iron homeostasis but also due to the potential cell signaling triggered by the unusual ceruloplasmin binding to integrin. Furthermore, there are pharmacological implications because the PD-CSF's pro-oxidant properties raise the possibility that in human, the proposed ceruloplasmin-based therapeutic approach might not be efficacious.

## Methods

### Patients and CSF samples

CSF samples were obtained from the Institute of Experimental Neurology-Biobank (San Raffaele Scientific Institute) after approval from the hospital’s ethical committee and informed consent from patients. The analyzed CFSs were from Parkinson’s disease patients (PD-CSF, *n* = 12), peripheral neuropathies patients (PN-CSF, *n* = 11) and healthy subjects (H-CSF, *n* = 16). All patients and controls were at first diagnosis and drug-free. Patients affected by probable idiopathic PD according to the current diagnostic criteria [40] were clinically evaluated by two expert neurologists and performed a thorough clinical-instrumental evaluation, including magnetic resonance imaging, in order to disclose possible differential diagnosis (i.e. vascular parkinsonism). The standard magnetic resonance imaging studies did not reveal region of focal atrophy in the patients. None of the PD patients presented behavioural disorders, including hallucinations or sleep disorders, at a thorough neurobehavioral and neuropsychological evaluation. Global cognitive efficiency was within normal range (Mini Mental State Examination ≥27/30) for all PD subjects with the exception of PD12. At the enrolment, all PD patients had short disease duration (<1 year from symptoms onset) with the exception of PD12. Motor system impairment and disease severity were determined in PD patients’ group by means of Unified Parkinson's Disease Rating Scale and Hoen and Yahr Stage. A clinical-instrumental follow-up after 1 year from the first hospitalization was done to confirm the diagnosis, only patients with confirmed PD diagnosis were included in the study and all presented a typical disease course.

A group of individuals affected by peripheral neuropathies was used as control group of different neurological disease. Current criteria for the diagnosis of PN were used for admission into the study [41]. Evaluation for disability was done according to Appel procedure; all patients underwent the normal procedure of differential diagnosis including haematological examination, electroneurographic and electromyographic evaluations, and CSF examination. At neurological examination all patients showed neurogenic changes and no motor or sensory conduction abnormalities. The disease course was chronically progressive in all cases. Patients were submitted to neurological examination using strength evaluation (MRC grading system) and Verbal-Numerical Pain Rating Scales performed at first hospitalisation and 6 and 12 months later, by the same neurologist.

Healthy CSFs were from subjects who underwent lumbar puncture on account of a suspected neurological disease and who proved to be free from pathological alterations after complete CSF analysis and clinic-neuroimaging assessment. All controls reported negative history of neurologic disorders and were normal on neurological examination. Exclusion criteria for all groups were: HIV/HCV-seropositivity; previous cerebral ischemic events; severe metabolic disorders. No statistical differences among all groups were found for sex distribution (Fischer’s exact test), for total protein concentration (Student’s *t*-test), and for patients age (H *vs.* PD and PD *vs.* PN groups comparison), whereas H *vs.* PN showed a *p* = 0.0317 (Mann–Whitney test). Demographic and clinical features of PD patients and control groups are summarized in Table 1 and Table 2, respectively.

**Table 1:** Demographic and clinical features of Parkinson’ disease patients

	Sex	Age at examination	[C] mg/ml	Diag.	Disease duration (months)	Clinical subtype	UPDRS I	UPDRS II	UPDRS III	Hoen & Yahr	MMSE
#1	M	65	0.38	PD	7	Tremble	0	4	4	1	30
#2	F	56	0.46	PD	10	Akinetic	1	9	29	1	29
#3	F	68	0.51	PD	8	Akinetic	2	3	14	2	28
#4	M	60	0.34	PD	10	Tremble	0	5	6	1.5	30
#5	M	70	0.39	PD	9	Tremble	0	3	5	1	30
#6	F	70	0.41	PD	12	Tremble	1	5	7	2	27
#7	F	73	0.46	PD	8	Tremble	3	9	19	1	29
#8	M	56	0.30	PD	-	Tremble	3	5	26	2	30
#9	F	66	0.60	PD	13	Tremble	0	8	17	1	30
#10	F	82	0.16	PD	9	Tremble	2	10	14	1	27
#11	M	80	0.47	PD	-	Akinetic	1	7	22	1.5	29
#12	F	56	0.19	PD + D	>12	Akinetic	7	20	34	2.5	22
	5 M/7 F	66.5 ± 8.3	0.39 ± 0.13								
		m ± sd	m ± sd								

[C] mg/ml = CSF total protein concentration; *UPDRS* = Unified Parkinson’s Disease Rating Scale; m = mean; sd = standard deviation

*PD* = Parkinson’s disease; *D* = dementia; Diag: confirmed diagnosis; *MMSE*: Mini Mental State Examination

**Table 2:** Demographic and clinical features of control groups

	H			PN			
	Sex	Age at examination	[C] mg/ml	Sex	Age at examination	[C] mg/ml	Diagnosis
#1	F	70	0.34	F	50	0.23	pnp-infl
#2	M	72	0.32	F	60	0.35	pnp-infl
#3	M	76	0.36	F	69	0.75	pnp-infl
#4	F	43	0.21	M	46	0.45	pnp-idio
#5	M	54	0.25	M	75	0.26	pnp-infl
#6	M	72	0.27	M	50	0.58	pnp-infl
#7	M	46	0.47	M	80	0.41	pnp-idio
#8	F	62	0.34	F	39	0.41	pnp-infl
#9	M	78	0.53	M	31	0.77	pnp-infl
#10	F	59	0.35	M	35	0.51	pnp-tox
#11	M	81	0.47	F	64	0.32	pnp-tox
#12	M	76	0.52				
#13	M	82	0.43				
#14	F	69	0.28				
#15	M	73	0.57				
#16	F	78	0.77				
	10 M/6 F	68.2 ± 12	0.41 ± 0.15	6 M/5 F	54.5 ± 16.4	0.46 ± 0.18	
		m ± sd	m ± sd		m ± sd	m ± sd	

*H* = healthy subjects; *PN* = peripheral neuropathies; [C] mg/ml = CSF total protein concentration; m = mean; sd = standard deviation; pnp-infl = peripheral neuropathy inflammatory; pnp-idio = pnp-idiopathic; pnp-tox = pnp-toxic

CSF samples (2–3 ml) were collected for bio-banking purposes by lumbar puncture and the first drops were discarded to avoid the artificial blood contamination. One ml of each CSF sample was made available for this study (2.5 ml for H1, PD10 and PD11 subjects). We set up a 40–50 min procedure for collection, processing and storage at −80 °C all CSF samples. Within this time frame samples remained at room temperature for 15–20 min for pneumatic tube transport, and at 4 °C for further 20–35 min during which the samples were centrifuged (800 x *g*, 10 min at 4 °C) to eliminate cells; then samples, were either immediately used or stored at −80 °C in a N_2_-supplemented atmosphere to avoid the exposure to oxidative-environment. No differences in results were observed in the different assays using fresh or stored CSF. For hydrogen peroxide quantification, the samples have been used within 6 months of storage and CSF aliquots were used only once to avoid repeated thawing/freezing cycles. CSF samples’ blood contamination was rule-out in all samples as no hemoglobin was detected by both WB and spectrophotometric analysis at 415 nm (see Additional File 1: Figure S1 and Table S1). The pH of the CSF from different groups was similar (H-CSF pH 8.58 ± 0.14, PN-CSF, pH 8.42 ± 0.32; and PD-CSF, pH 8.61 ± 0.12) and consistent with the reported values for collected CSF [42]. The pH reached 9.65 ± 0.01 in all groups at the end of the *ex vivo* incubation time (see below). Due to the limited amount of CSF samples available, and depending on the sample’s volume necessary for the different assays, not all the patients and controls were used in each experiment; numbers of subjects used in the different assays are indicated in the figure legends.

### Incubation treatments of ceruloplasmin in CSFs and immunoprecipitation

Purified human ceruloplasmin (Alexis Biochemicals) was added to CSFs (20 μg/ml, final concentration) and incubated at 37 °C to the indicate time points under nitrogen-conditioned atmosphere, to avoid the exposure to atmospheric oxidative environment; in selected experiments, incubation was performed in presence of catalase (60 μg/ml, Sigma-Aldrich). After incubation in CSFs, spiked ceruloplasmin was immunoprecipitated using protein-G agarose beads (Invitrogen) coated with polyclonal sheep anti-ceruloplasmin antibody (Ab) (ab8813 Abcam, raised against full length purified ceruloplasmin) cross-linked with 20 mM dimethyl-pimelimidate (Sigma-Aldrich). This polyclonal Ab anti-ceruloplasmin was selected for its ability to equally immunoprecipitate both resting- and oxidized-ceruloplasmin (see Additional File 1: Figure S2). Ceruloplasmin was eluted with 0.1 M glycine, pH 2.5, and diafiltered (Amicon-Millipore) in PBS. *In vitro* oxidation and deamidation of ceruloplasmin (Cp-ox/AmBic) was achieved by incubation (16 h at 37 °C) in 100 mM ammonium bicarbonate buffer pH 8.5, containing 10 mM hydrogen peroxide [14].

### Ceruloplasmin binding to αvβ6 integrin and competition with isoDGR peptide

Recombinant αvβ6 integrin (R&D Systems, 1 μg/ml in PBS-Ca^2+^/Mg^2+^) was added to 96-well plates and incubated 16 h at 4 °C; subsequent steps were carried out at 20 °C. After blocking with 3 % BSA-PBS, the plates were filled (50 μl/well 1:1 in *binding buffer,* 25 mM Tris–HCl, pH 7.4, 150 mM NaCl, 1 mM MgCl_2_, 1 mM MnCl_2_, 0,05 % Tween, 1 % BSA) with CSF samples either from healthy or pathological subjects, or in selected experiments with CSF samples previously supplemented with ceruloplasmin or with Cp-ox/AmBic and incubated for 2 h. Binding was detected using the sheep polyclonal anti-human ceruloplasmin Ab (ab8813, Abcam) followed by a secondary HRP-conjugate Ab (Abcam) and by o-phenylendiamine chromogenic substrate. Competitive binding assays were performed by mixing 30 μg/ml of either the acetyl-CisoDGRCGVRSSSRTPSDKY peptide (isoDGR-peptide) or the control peptide CARACGVRSSSRTPSDKY (ARA-peptide) [43] with CSF samples spiked with ceruloplasmin.

### Mass spectrometry analysis

#### Analysis on spiked-in ceruloplasmin

For MS analysis, we used a protocol set up as to avoid experimental protein oxidation and/or deamidation [14]. In brief, ceruloplasmin eluted from the immunoprecipitation was diafiltered in *digestion buffer* (50 mM Na_3_PO_4_ pH 7.3, 150 mM NaCl, 25 mM HEPES) and incubated with trypsin (0.1 μg/μl, Roche Diagnostics) for 2 h at 20 °C. Samples were desalted (Stage tips C18, Thermo Scientific) and injected in a capillary chromatographic system (EasyLC, Proxeon Biosystem). Peptide separations occurred on a 25 cm reverse phase silica capillary column, packed with 3-μm ReproSil-Pur 120 C18-AQ. A gradient of acetonitrile eluents was used to achieve separation (0.15 μL/min flow rate). MS analysis was performed by nanoLC-MS/MS using an LTQ-Orbitrap (Thermo Scientific) equipped with a nano-electrospray ion source (Proxeon Biosystems). Full scan spectra were acquired with the lock-mass option, resolution set to 60,000, and mass range from *m*/*z* 300 to 1750 Da. The ten most intense doubly and triply charged ions were selected and fragmented in the ion trap. All MS/MS samples were analyzed using Mascot (v.2.2.07, Matrix Science) search engine to search the UniProt_Human Complete Proteome_cp_hum_2013_12. Searches were performed with 3-missed cleavages allowed, N-terminus-acetylation, methionine oxidation and deamidation of asparagine/glutamine as variable modifications. Mass tolerance was set to 5 ppm and 0.6 Da for precursor and fragment ions, respectively. To quantify deamidation the raw data were loaded into the MaxQuant software v1.3.0.5. Label-free protein quantification was based on the intensities of precursors. Peptides and proteins were accepted with a FDR of 0.01, two minimum peptides per protein with one unique peptide.

#### Analysis on endogenous ceruloplasmin

CSF samples (70 μl each) were diluted (1:2) with *digestion buffer* and incubated 12 h at 20 °C in the presence of trypsin 1:10 (w/w). The tryptic mixture was desalted and injected in a capillary chromatographic system (EASY-nLC™ 1000 Integrated Ultra High Pressure Nano-HPLC System, Proxeon Biosystem) for peptide separations on a 10 cm reverse phase silica capillary column, packed with 1.9-μm ReproSil-Pur 120 C18-AQ. A 60 min-gradient of acetonitrile was used to achieve separation (0.30 μL/min flow rate). Parallel reaction monitoring (PRM) analyses were performed using a Q-Exactive mass spectrometer (Thermo Scientific). The acquisition method combined two scan events corresponding to a full scan MS (resolution set to 35,000 at m/z 200) and a PRM event (resolution set to 17,500 at m/z 200; isolation window set to 2 m/z; maximum fill time of 120 ms and normalized collision energy set to 27) which targeted the precursor ions of the peptides at their relevant charge states in 1 min window. Using trypsin-digested purified Cp and Cp-ox/AmBic, a local spectral library was created to generate reference MS/MS spectra (b- and y-fragment ions) and to determine the elution time for each peptide. Data processing was performed with Skyline software, freely available [44]. The quantification was performed, post-acquisition, by extracting the chromatographic traces of specific fragment ions, from 3 to 5 transitions. For each targeted peptide, extracted ion chromatograms were selected for three to five transitions. Peak areas, calculated from the sum of transitions (excluding the precursors), were automatically estimated by Skyline software. Internal reference peptides (IRPs) were selected as unmodified proteotypic peptides with good signal stability and a wide range of elution times. The sum of transition peak areas for each peptide was divided either by the sum of transition peak areas for individual reference Cp peptides or by the sum of the transition peak areas for all reference Cp peptides [45]. The use of the Cp reference peptide LISVDTEHSNIYLQNGPDR yielded the lowest median CV (10.7 %) for both ^962^NGR- and ^962^DGR-containing peptides. Five technical replicates for each sample were run.

##### Western blot

Ceruloplasmin immunoprecipitated from CSF after incubation was resolved by 10 %-acrylamide SDS-PAGE, and analyzed by WB as described in [3] with the polyclonal sheep anti-human ceruloplasmin Ab (ab8813, Abcam) followed by a secondary HRP-conjugate Ab (Abcam); images were acquired using a laser densitometer (Molecular Dynamics).

##### Bathophenanthroline assay

Ferroxidase activity of spiked ceruloplasmin after aging in CSF was evaluated by bathophenanthroline (Btp) assay as described in [3] on the immunoprecipitated ceruloplasmin. Briefly, 1.25 μg of immunoprecipitated ceruloplasmin was incubated with 80 mM FeSO_4_ and analysed after 1 h with a solution of 1 mM Btp in acetate buffer, pH 6.2. Decrease in Btp-Fe^2+^ complex absorbance at 535 nm derives from ferrous iron oxidation into ferric form (Fe^3+^). Ferroxidase activity was reported as percentages of the activity recorded for 1.25 μg of untreated ceruloplasmin.

##### Ceruloplasmin sensitivity to limited proteolysis

Spiked ceruloplasmin after aging in CSF was immunoprecipitated from CSF and 100 ng were resuspended in 25 μl of *digestion buffer* followed by incubation for 2 h at 20 °C in the presence of agarose-immobilized trypsin (0.5 U) (Sigma-Aldrich). Trypsin was removed by centrifugation, and samples were analysed by SDS-PAGE and WB.

##### Carbonylation assay

The carbonylation level of ceruloplasmin immunopreciptated from CSF after aging, was analyzed as in [3] with the OxyBlot Protein Oxidation Detection Kit (Millipore Bioscience) on the basis of carbonyl group derivatization with 2,4-dinitrophenilhydrazine (DNP). After derivatization with DNP, ceruloplasmin (300 ng) was resolved by SDS-PAGE, and carbonyl groups were detected by WB using an anti-DNP Ab.

##### Hydrogen peroxide assay

Hydrogen peroxide concentration in the CSFs was evaluated using the Hydrogen Peroxide Assay Kit (BioVision) that is based on the reaction between the H_2_O_2_ and a non-fluorescent probe (AmplexRed®) that produces a fluorescent compound. The analysis was performed on either freshly collected CSF or samples stored at −80 °C for less than 6 months and that were not previously thawed. Samples were analyzed in triplicate diluting CSF 1:100 in the assay buffer according to the manufacturer protocol, and H_2_O_2_ concentration was evaluated as fluorescence at 590 nm.

##### Statistical Analysis

Sex distribution was analyzed by using Fisher’s exact test and two-tailed p value. Continuous data were evaluated by unpaired student’s *t*-test, if the data passed the normality test for Gaussian distribution as assessed by the Kolmogorov-Smirnov test, or were evaluated by Mann Whitney test; two-tailed p value was used for the comparison of two means and standard error. In all analyses, p < 0.05 was considered to be statistically significant. The analysis was performed with GraphPad Prism V5 software.

## Additional files

Additional file 1:
**Figure S1.** Western blot analysis for contaminant hemoglobin detection. **Figure S2.** Immunoprecipitation of both resting- and oxidized-ceruloplasmin.**Table S1.** Spectrophotometric analysis for hemoglobin contaminant detection in CSF samples. (PDF 1445 kb)

## Abbreviations

Cp: Ceruloplasmin
CSF: Cerebrospinal fluid
PD: Parkinson’s disease
Asp or D: Aspartate
*iso*Asp or *iso*D: Isoaspartate
NGR: Asparagine-Glycine-Arginine
RGD: Arginine-Glycin-AsparticAcid
MS: Mass spectrometry
PRM: Parallel reaction monitoring
H: Healthy
PN: Peripheral neuropathies
WB: Western blot
Cp-ox/AmBic: *in-vitro* oxidized/deamidated ceruloplasmin
Ab: Antibody
IRP: Internal reference peptide
Btp: Bathophenanthroline
DNP: 2,4-dinitrophenilhydrazine
