# Supplementary material for: Ceruloplasmin functional changes in Parkinson’s disease-cerebrospinal fluid
Source: Mol Neurodegener. 2015 Nov 4;10:59. doi: 10.1186/s13024-015-0055-2 (PMC4634150; doi:10.1186/s13024-015-0055-2)

Ceruloplasmin functional changes in Parkinson's disease-cerebrospinal fluid

Marco Barbariga, Flavio Curnis, Annapaola Andolfo, Alan Zanardi, Massimo Lazzaro, Antonio Conti, Giuseppe Magnani, Maria Antonietta Volontè, Laura Ferrari, Giancarlo Comi, Angelo Corti, Massimo Alessio

Supplementary Figure 1

Western blot (WB) analysis for contaminant hemoglobin detection

Blood contamination of CSF samples was assessed by WB performed with goat polyclonal anti-hemoglobin antibody (Santa Cruz biotechnology, SC-31110) on 30 µl of CSF from each samples resolved by 12% SDS-PAGE. Different amounts (7, 20, 70 µg, total protein) of a standard hemolyzed serum were used in all the experiments as reference for contaminant hemoglobin detection.

A representative result is shown:

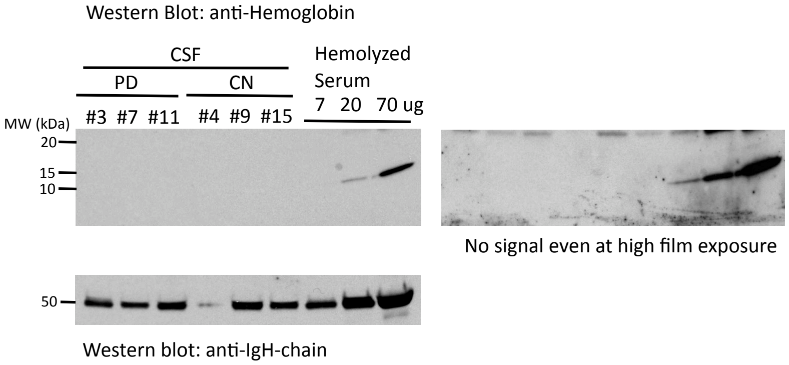

Supplementary Table 1

Spectrophotometric analysis for hemoglobin contaminant detection in CSF samples

Spectrophotometric analysis was performed by multiwavelenght determination scanning in continuous absorbance spectra (200-600 nm) of the CSF samples in 70 µl microcuvettes with a 1 cm light path, using the Ultrospec 5300pro spectrophotometer (GE-Healthcare). Quantitative hemoglobin measurement was done recording the absorbance at 415 nm wavelength, the specific peak of absorbance for hemoglobin (Smith A. et al. Clin Chim Acta, 424: 231-6, 2013). A standard control curve was

generated adding decreasing amounts of hemolysed red blood cell (2, 1, 0.5, 0.25, 0.125, 0 μg of total protein) to the CSF from a healthy subject. All the CSF samples used in the study showed no-absorbance peak at 415 nm (absorbance values of about 0.200 A.U. or lower), which in turn indicated the absence of hemoglobin contamination.

Hemoglobin measurements by spectrophotometry absorbance at 415 nm wave length

Standard curve	Absorbace (A.U.)	subject	**H-CSF[a]**	**PN-CSF[a]**	**PD-CSF[a]**
CSF+RBC (2 μg)	2.8250	#1	0.1960	0.1900	0.1557
CSF+RBC (1 μg)	1.4530	#2	0.1660	0.1653	0.1590
CSF+RBC (500 ng)	0.8400	#3	0.2010	0.1997	0.1557
CSF+RBC (250 ng)	0.5310	#4	0.1687	0.1983	0.1617
CSF+RBC (125 ng)	0.3650	#5	0.1973	0.1983	0.1553
CSF	0.1950	#6	0.1637	0.1640	0.1560
		#7	0.1887	0.1867	0.1817
		#8	0.1947	0.1613	0.1540
		#9	0.1677	0.1957	0.1593
		#10	0.2017	0.1930	0.1623
		#11	0.2000	0.1900	0.1573
		#12	0.1683		0.1610
		#13	0.1927		
		#14	0.1933		
		#15	0.1660		
		#16	0.2003		

A.U., arbitrary units; RBC,hemolysed red blood cells total proteins; H-CSF,cerebrospinal fluid from healthy subjects;
PN-CSF, cerebrospinal fluid from peripheral neuropathies patients; PD-CSF, cerebrospinal fluid from Parkinson's disease patients
a, absorbance at 415 nm (arbitrary units).

Supplementary Figure 2

Immunoprecipitation of both resting- and oxidized-ceruloplasmin

Cerebrospinal fluid from a healthy subject, either under resting conditions or after oxidation treatment with 10 mM hydrogen peroxide (16 h at 37°C) was used to test the immunoprecipitation capability of different anti-ceruloplasmin antibodies. After hydrogen peroxide or mock incubation treatments, ceruloplasmin (Cp) was immunoprecipitated from the CSF using protein-G agarose beads (Invitrogen) coated with different antibodies (Ab); mouse anti-Cp (sc21240, Santa Cruz Biotech, raised against the human ceruloplasmin N-terminus); rat anti-Cp (sc21242, Santa Cruz

Biotech raised against the human ceruloplasmin C-terminus); polyclonal sheep anti-ceruloplasmin antibody (ab8813, Abcam raised against full length purified Cp). Abs were cross-linked to agarose beads with 20 mM dimethyl-pimelimidate (Sigma). After immunoprecipitation ceruloplasmin was eluted with 0.1 M glycine, pH 2.5, and analysed by Western blot. The polyclonal Ab anti-ceruloplasmin was selected for the following studies due its ability to equally immunoprecipitate both resting- and oxidized-ceruloplasmin.

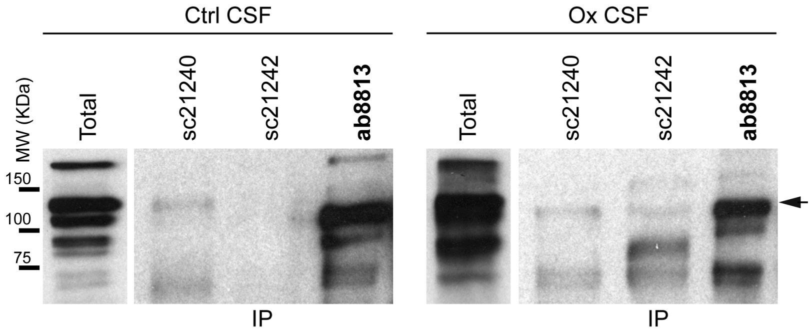

Supplement: Additional file 1: — Figure S1. Western blot analysis for contaminant hemoglobin detection. Figure S2. Immunoprecipitation of both resting- and oxidized-ceruloplasmin.Table S1. Spectrophotometric analysis for hemoglobin contaminant detection in CSF samples. (PDF 1445 kb) [file 13024_2015_55_MOESM1_ESM.pdf]
